# Prolonged survival after thoracic metastasectomy in patients with nonseminomatous testicular cancer

**DOI:** 10.1016/j.clinsp.2024.100338

**Published:** 2024-02-15

**Authors:** Jaqueline Schaparini Fonini, Pedro Henrique Xavier Nabuco de Araujo, Paula Duarte D'Ambrosio, Juliana Vieira de Oliveira Salerno, Pedro Prosperi Desenzi Ciaralo, Ricardo Mingarini Terra, Paulo Manuel Pêgo-Fernandes

**Affiliations:** a Thoracic Surgery Department, Hospital das Clínicas, Faculdade de Medicina, Universidade de São Paulo (HCFMUSP), São Paulo, SP, Brazil; b Thoracic Surgical Oncology, Hospital das Clínicas, Faculdade de Medicina, Universidade de São Paulo (HCFMUSP), São Paulo, SP, Brazil

**Keywords:** Nonseminomatous germ cell tumor, Chemotherapy, Metastasis, Teratoma, Thoracic surgery, Mortality

## Abstract

**Introduction:**

Almost 20 % of patients with Non-Seminomatous Germinative Cell Tumors (NSGCT) will require intrathoracic metastasectomy after chemotherapy. The authors aim to determine their long-term survival rates.

**Methods:**

Retrospective study including patients with NSGCT and intrathoracic metastasis after systemic therapy from January 2011 to June 2022. Treatment outcomes and overall survival were analyzed with the Kaplan-Meier method.

**Results:**

Thirty-seven male patients were included with a median age of 31.8 years. Six presented with synchronous mediastinum and lung metastasis, nine had only lung, and 22 had mediastinal metastasis. Over half had retroperitoneal lymph node metastasis. Twenty-two had dissimilar pathologies, with a discordance rate of 62 %. Teratoma and embryonal carcinoma were the prevalent primary tumor types, 40.5 % each, while teratoma was predominant (70.3 %) in the metastasis group. Thoracotomy was the main surgical approach (39.2 %) followed by VATS (37.2 %), cervico-sternotomy (9.8 %), sternotomy (5.8 %), and clamshell (3.9 %). Lung resection was performed in 40.5 % of cases. Overall, 10-year survival rates were 94.3 % with no surgical-related mortality.

**Conclusion:**

Multimodality treatment with systemic therapy followed by radical surgery offers a high cure rate to patients with intrathoracic metastatic testicular germ cell tumors.

## Highlights

• Few studies have examined outcomes after intrathoracic metastasectomy for nonseminomatous testicular germ cell tumors.
• Integration of local and systemic therapies yields favorable outcomes with low morbidity and mortality.
• Surgical management should be considered after chemotherapy, as histology prediction for metastatic lesions remains challenging.
• This study highlights the importance of aggressive surgical approaches in improving long-term survival in young patients with testicular NSGCT.

## Introduction

Nonseminomatous Germ Cell Tumors (NSGCTs) of testicular origin are the most common neoplasm in male patients younger than 40 years of age.^1^ It is estimated that 8000 new cases are diagnosed in the United States each year. Approximately half of these patients present with metastasis at diagnosis.^2^ Even though testicular NSGCT may produce hematogenous lung, brain, and bone metastasis, it is more frequent to have lymphatic metastasis to the mediastinum and retroperitoneum, and the majority of patients with supradiaphragmatic disease are cured with chemotherapy alone.

Although the incidence of testis cancer is rising, NSGCT currently represents one of the most successful models for multimodality cancer therapy.^3^^,^^4^ An estimated 10–20 % of patients with advanced metastatic disease previously treated with chemotherapy present extra-testicular tumors that require mediastinal dissection or pulmonary metastasectomy.^5^ The most significant surgical studies have shown that residual masses contain viable tumor cells and teratoma in 12 % to 15 % and 34 % to 42 % of cases, respectively.^5^, ^6^, ^7^, ^8^, ^9^ Although teratomas are benign by definition, they have the potential to transform into other types of somatic malignancies even after chemotherapy, posing a persistent risk if left untouched.^10^ Complete necrosis is found in residual lung masses in a relatively high proportion of patients, ranging from 54 % to 71 % after normalization of serum tumor markers post-chemotherapy.^8^^,^^9^^,^^11^ Currently, there is no differentiation between necrosis and teratoma preoperatively, rendering surgery the only option for diagnostic purposes.

Moreover, comparative pathology analysis of Retroperitoneal Lymph Node Dissection (RPLND) specimens and lung metastasis has unveiled discordance between lesion components in 28 % to 36 % of cases.^8^^,^^9^^,^^11^ Extensive resection of all residual masses is therefore recommended by most authors. However, in this setting, surgical complications range from 6 % for an isolated lung metastasis and 8 % for an isolated RPLND to 13 % for mediastinal resection or 35 % for a sequential RPLND and thoracic resection.^12^^,^^13^

Long-term outcome data after post-chemotherapy RPLND has been widely reported, however, few studies have shown the clinical outcomes in patients with testicular NSGCT treated with intrathoracic metastasectomy.^4^ The purpose of this retrospective study was to determine the long-term survival rates of patients with testicular germ cell tumors undergoing intrathoracic metastasectomy after chemotherapy.

## Material and methods

A retrospective descriptive analysis was performed from a prospective database including all patients who had resection of intrathoracic metastatic NSGCT after chemotherapy between January 2011 and June 2022 at Instituto do Cancer do Estado de São Paulo (ICESP), Hospital das Clinicas HCFMUSP, Faculdade de Medicina, Universidade de São Paulo, São Paulo, SP, BR. The study was approved by the Research Ethics Board (33,365,720.2.0000.0068). The need for written informed consent was waived due to the study's retrospective and noninterventional nature. Patients with primary mediastinal NSGCT were excluded due to the different treatment protocols and those who did not undergo chemotherapy or intrathoracic metastasis resection. The variables recorded when available were age, sex, metastasis location, number of surgical procedures, histology, surgical access types, complications, retroperitoneal lymph node resection, risk classification according to the International Germ-Cell Cancer Collaborative Group (IGCCCG), and disease-free survival period. The pathologies of the primary tumor were viable germ cells, somatic malignancy, teratoma, and necrosis. Postoperative complications were classified according to the Common Terminology Criteria for Adverse Events Version 5 (CTCAE V5).^14^

Demographic data were reported as median and range, and a descriptive analysis of the categorical variables was performed. Survival rates were estimated using the Kaplan-Meier method, based on the date of the first thoracic resection.

## Results

Over the study period, 37 patients underwent intrathoracic resection of residual post-chemotherapy mediastinal masses after radical inguinal orchiectomy. All patients were men, and the mean age was 31.8 (range ± 7.5). Six patients (16.2 %) presented with synchronous mediastinum and lung metastasis, nine patients had only lung metastasis (24.3 %) and 22 patients had exclusive mediastinum metastasis (59.5 %). When classified based on the IGCCCG risk category, 18 patients were considered a good risk (48.7 %), 6 patients were intermediate risk (16.2 %) and 13 patients were poor risk (35.1 %) as presented in Table 1.

**Table 1:** Clinical characteristics of patients with non-seminomatous germinative cell - entire cohort.

**Sex, n (%)**		
Male	37	100.0
Female	0	0.0
		
**Median age (range)**	31.8 (±7.5)	
		
**Site of resection**		
Mediastinum	22	59.5
Lung	9	24.3
Mediastinum + Lung	6	16.2
		
**Initial IGCCCCG^a^ risk, n (%)**		
Good	18	48.7
Intermediate	6	16.2
Poor	13	35.1

(*n* = 37).

^a^IGCCCG, International Germ Cell Cancer Collaborative Group.

**Fig. 1:**
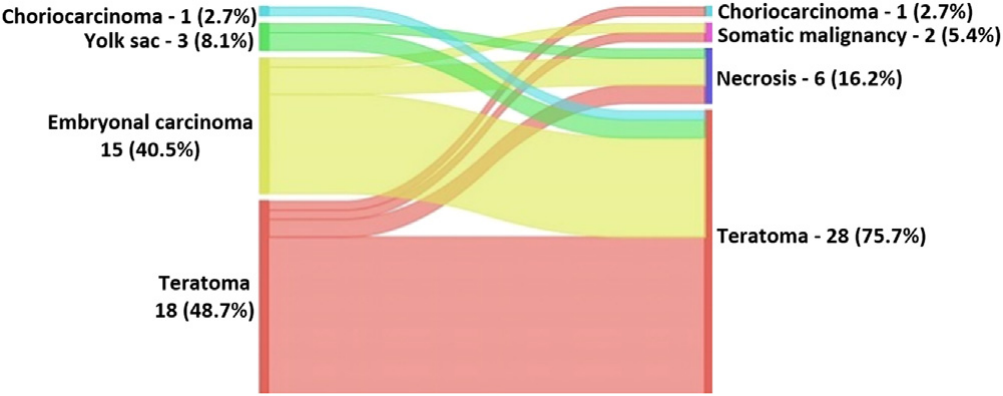
Primary tumors are represented on the left and metastasis in the right. (*n* = 37 patients). Color code: Choriocarcinoma ‒ light blue; Yolk sac ‒ green; Embryonal carcinoma ‒ yellow; Teratoma ‒ red; Somatic malignancy ‒ pink; Necrosis ‒ dark blue.

Histologic examination of the metastasectomy specimens revealed necrosis in 6 patients (16.2 %), teratoma in 26 patients (70.3 %), and viable carcinoma was found in 2 patients (one pure embryonal carcinoma and one choriocarcinoma). Nonetheless, most patients had discordant primary and metastatic pathologies (62.2 %). Teratoma was the prevalent histology in both primary (48.6 %) and metastatic (37.8 %) tumors. Of 15 patients with primary histology of embryonal carcinoma, eleven (73.3 %) had metastasis compatible with teratoma, as well as 2 of the 3 patients with yolk sac primary histology and one patient with choriocarcinoma (Fig. 1).

Unilateral thoracotomy was the most frequently used surgical approach (39.2 %), followed by Video-Assisted Thoracic Surgery (VATS) (37.2 %), cervico-sternotomy (9.8 %), sternotomy (5.8 %), and clamshell (3.9 %). Two patients were submitted to cervicotomy, however, one of them had complementary mediastinal tumor resection by VATS. Patients who underwent the clamshell approach had metastasis only in the mediastinum and did not need other surgical interventions.

There were 26 patients (70.3 %) who had one surgical procedure, 8 patients (21.6 %) had two surgical procedures and 3 (8.1 %) were submitted to three surgical procedures after chemotherapy due to the slow progression of the lymph node disease. Additionally, pulmonary resection was performed in 15 patients (40.5 %). Planned, sequential, or stage interventions were not considered as reoperations. More than half of the patients (64.8 %) also had retroperitoneal lymph node metastasis. Eighteen patients (48.6 %) underwent intrathoracic surgical procedures after RLND. In contrast, 6 patients (16.2 %) were submitted to intrathoracic metastasectomy before the RLND, and 13 patients (35.1 %) did not require any RLND (Table 2).

**Table 2:** Surgical approach for intrathoracic metastasectomy.

**Surgical approach, n (%)**		
Cervicotomy	2	(3.9)
Cervicotomy + Sternotomy	5	(9.8)
Thoracotomy	20	(37.2)
Videothoracoscopy	19	(39.2)
Sternotomy	3	(3.9)
Clamshell	2	(5.8)
**TOTAL**	**51**	**(100.0)**
		
**Total number of thoracic resections, n (%)**		
1	26	(70.3)
2	8	(21.6)
3	3	(8.1)
**Lung resection, n (%)**		
Yes	15	(40.5)
No	22	(59.5)
**TOTAL**	**37**	**(100.0)**
		
**Retroperitoneal lymph node dissection**	**n (%)**	
Before mediastinum	18	(48.6)
After mediastinum	6	(16.2)
None	13	(35.1)
**TOTAL**	**37**	**(100.00)**

**Fig. 2:**
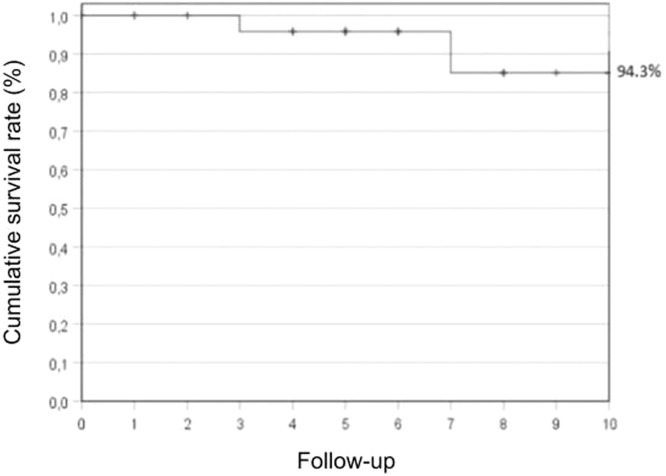
Survival rate after pulmonary or mediastinal resection with 10-year follow-up. Database = 37-patients.

Operative mortality was low among these young male patients, with no surgical deaths. Two patients died after over five years of the surgical procedure, one of renal failure due to disease progression and chemotherapy toxicity, and the other one died of renal failure due to late complications of abdominal trauma surgery, not related to the intrathoracic metastasis resection. After a 10-year follow-up, the overall survival rate was 94.3 % (Fig. 2), and there was a 5-year median survival rate. Forty percent of all patients who underwent intrathoracic metastasectomy were disease-free for 4 to 6 years after surgery, and 25 % had 7 to 10 years with no recurrence.

Four patients had nonfatal postoperative complications. According to the CTCAE V5, one had a Grade 3 complication due to a bilateral phrenic nerve injury and needed a temporary tracheostomy. The other three were Grade 2 complications, including a bronchopleural fistula with no hemodynamic repercussions and no surgical intervention required, and two superficial wound infections.

## Discussion

In our results, teratoma and embryonal carcinoma were the most prevalent primary tumor histology, representing 40.5 % each, but in the metastasis group, teratoma was the most frequent cell type (70.3 %). The germ-cell types comprise embryonal carcinoma, teratoma, choriocarcinoma, and yolk-sac carcinoma. The first is the most undifferentiated cell type, with the capacity to differentiate from the others.^12^ About 8.3 % of all intrathoracic metastasectomy (pulmonary and mediastinal) contain viable GCT, and the remainder contains either necrosis/fibrosis or mature teratoma in 91.7 %. Overall 10-year survival rates were 94.3 % with no surgical-related mortality, despite four patients having nonfatal postoperative complications, including bilateral phrenic nerve injury, bronchopleural fistula, and two superficial wound infections. The high cure rates have generated a strong bias toward surgical resection of any residual lesions after chemotherapy in patients with these cancers.

After effective chemotherapy, aggressive surgical resection to remove the residual tumors in selected patients with intrathoracic metastasis has proven to increase disease-free survival rates, this integrated approach is in complete agreement with our results.^15^ The overall 10-year survival rate in our study was 94 % with no surgical-related mortality and few postoperative complications. Other authors report a similar experience on the outcomes of patients with testicular NSGCT with resection of residual intrathoracic disease after cisplatin-based chemotherapy: Liu and colleagues and Cagini and colleagues published a previous series consisting of patients with testicular NSGCT who underwent intrathoracic metastasectomy of pulmonary and/or mediastinal metastasis, reporting 5 year survivals of 71 % and 77 %, respectively.^13^^,^^16^ Certainly, those higher survival rates also could be attributed to the appropriate patient selection with an individualized surgical strategy, multidisciplinary approach, and careful surgical planning.

In contrast, the treatment of patients with residual masses after chemotherapy is controversial because the histology of the resected specimen is often revealed to be necrosis. Thus, it is unclear whether or not resection of all lesions is necessary, and watchful waiting may be an option. However, persistent radiographic residual masses accompanied by normalized tumor marker levels in the serum can contain necrotic tissue alone, differentiated teratoma, or undifferentiated tumors with viable tumor cells.^5^ Several studies have attempted to predict the histology of the residual lesions preoperatively to avoid surgery in those patients with benign residual mass, yet to date, no parameter has been established as a reliable predictor.^5^^,^^13^^,^^17^^,^^18^

At least 33 % of suspected metastatic lesions have a different histology from the primary tumor.^6^^,^^19^ In our results, 22 of 37 residual metastases had dissimilar pathologies, with a discordant rate of 62 %. Teratoma and embryonal carcinoma were the most prevalent primary tumor types, representing 40.5 % each, but in the metastasis group, teratoma was by far the predominant cell type (70.3 %). Only 8.3 % of all intrathoracic metastasis (pulmonary and mediastinal) contained viable GCT, the rest represented either necrosis/fibrosis or mature teratoma (91.7 %). Surgical resection of all viable sites of metastatic disease was performed in our group of patients and postoperative management depended on the final pathology of all resected masses. No further treatment was needed for necrosis/fibrosis and mature teratomas.

Commonly, multiple surgical procedures are required to remove bilateral or multiple levels of residual mediastinal disease or disease that presents during long-term follow-up.^12^^,^^15^ In this series, 29.7 % of the patients had more than one surgical procedure and still achieved a long survival rate with no operative mortality. Kesler and colleagues also reported an excellent long-term survival rate (78 %) with the removal of residual mediastinal disease after chemotherapy over 400 thoracic surgical procedures in nearly 300 patients with NSGCT.^12^ Because operative morbidity and mortality rates were low (1 %) with prolonged survival being possible in these otherwise young and healthy patients, aggressive thoracic surgical management and timely repeat surgical intervention are justified in select patients.^12^

The present study has several limitations, including its retrospective nature, single-center design, and small sampling due to the rarity of the disease. Also, the authors did not include information about adjuvant chemotherapy used and as the present study's hospital is a referral oncology center, it might not be generalizable. However, no pre-surgical algorithm to this date has proven to be effective at predicting metastasis histologic outcome, and similar to that in pulmonary metastasectomy in general, no prospective randomized trials have been conducted to define the role of surgery versus a nonsurgical treatment regimen. The survival advantage conferred by surgical thoracic disease control for germ-cell tumors is favorable, as shown in this study. In addition, surgical resection is already routinely a part of the multimodal management of these patients, which makes the need for further prospective randomized studies redundant.

## Conclusions

Intrathoracic metastasectomy in testicular NSGCT illustrates the curative potential of integrating local and systemic therapy with low morbidity and mortality rates, even in cases where more than one surgery is required. Given the low concordance between primary and metastatic disease and no means of prediction of the histology of the latter, aggressive surgical management should be considered after chemotherapy due to the potential benefit in long-term survival in these otherwise young patients.

## Synopsis for table of contents

Multimodality treatment with systemic therapy followed by radical surgery offers a high cure rate for patients with intrathoracic metastatic testicular germ cell tumors. The long-term survival rates establish its benefit and safety and should always be considered as a viable treatment in this group of patients.

## CRediT authorship contribution statement

**Jaqueline Schaparini Fonini:** Investigation, Writing – original draft, Writing – review & editing. **Pedro Henrique Xavier Nabuco de Araujo:** Conceptualization, Project administration, Writing – review & editing. **Paula Duarte D'Ambrosio:** Methodology, Validation, Formal analysis, Writing – review & editing, Visualization. **Juliana Vieira de Oliveira Salerno:** Investigation, Writing – original draft, Writing – review & editing, Visualization. **Pedro Prosperi Desenzi Ciaralo:** Conceptualization, Methodology. **Ricardo Mingarini Terra:** Supervision, Writing – review & editing. **Paulo Manuel Pêgo-Fernandes:** Supervision.

## Conflicts of interest

The authors declare no conflicts of interest.

## Appendix Supplementary materials

Image, application 1
