# Supplementary figures and images for: Prolonged survival after thoracic metastasectomy in patients with nonseminomatous testicular cancer
Source: Clinics (Sao Paulo). 2024 Feb 15;79:100338. doi: 10.1016/j.clinsp.2024.100338 (PMC10877677; doi:10.1016/j.clinsp.2024.100338)

CLINICS-D-23-00559_Supplementary material

**Visual Abstract**

**
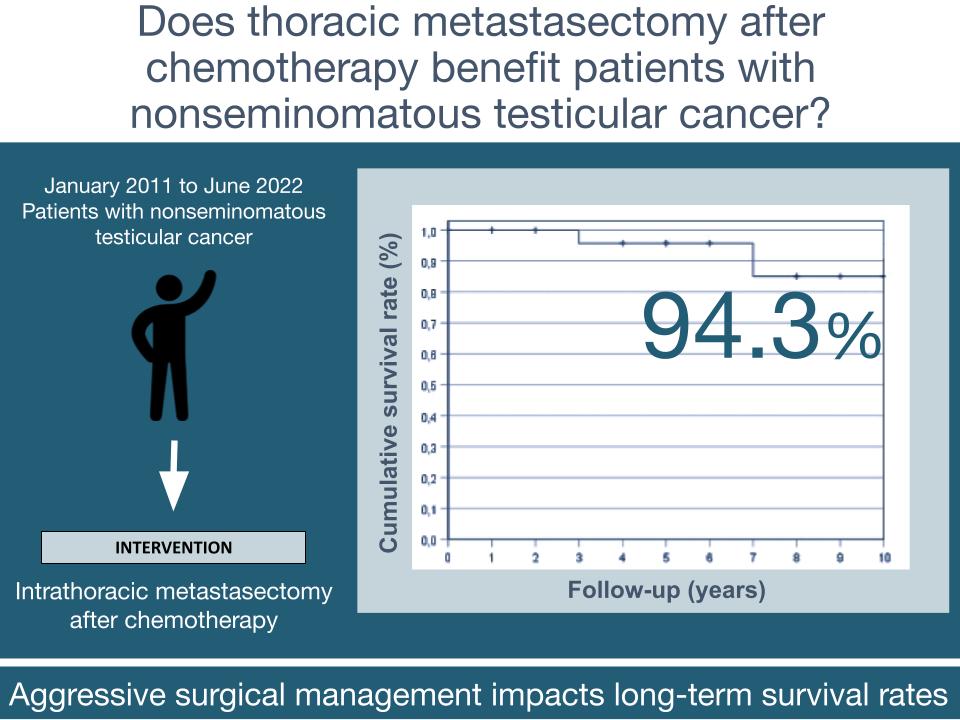
**

Supplement: Supplementary file 1 [file mmc1.docx]
